# Targeting Immune Cell Trafficking – Insights From Research Models and Implications for Future IBD Therapy

**DOI:** 10.3389/fimmu.2021.656452

**Published:** 2021-05-04

**Authors:** Maximilian Wiendl, Emily Becker, Tanja M. Müller, Caroline J. Voskens, Markus F. Neurath, Sebastian Zundler

**Affiliations:** ^1^ Department of Medicine 1, Deutsches Zentrum Immuntherapie (DZI), University Hospital Erlangen, Friedrich-Alexander-Universität Erlangen-Nürnberg, Erlangen, Germany; ^2^ Department of Dermatology, University Hospital Erlangen, Friedrich-Alexander-Universität Erlangen-Nürnberg, Erlangen, Germany

**Keywords:** IBD, T cell, trafficking, homing, retention, therapy

## Abstract

Inflammatory bowel diseases (IBDs), including Crohn’s disease (CD) and ulcerative colitis (UC) ****are multifactorial diseases with still unknown aetiology and an increasing prevalence and incidence worldwide. Despite plentiful therapeutic options for IBDs, the lack or loss of response in certain patients demands the development of further treatments to tackle this unmet medical need. In recent years, the success of the anti-α4β7 antibody vedolizumab highlighted the potential of targeting the homing of immune cells, which is now an important pillar of IBD therapy. Due to its complexity, leukocyte trafficking and the involved molecules offer a largely untapped resource for a plethora of potential therapeutic interventions. In this review, we aim to summarise current and future directions of specifically interfering with immune cell trafficking. We will comment on concepts of homing, retention and recirculation and particularly focus on the role of tissue-derived chemokines. Moreover, we will give an overview of the mode of action of drugs currently in use or still in the pipeline, highlighting their mechanisms and potential to reduce disease burden.

## Introduction

Trafficking of immune cells, including T lymphocytes, to the gut is a tightly regulated multistep process important for maintaining homeostasis and initiating immune responses (1–4). Naïve T cells circulate through secondary lymphoid organs until they encounter their cognate antigen presented by dendritic cells (DCs) in the gut-associated lymphoid tissue (GALT). This interaction leads to activation, proliferation and imprinting of T cells with a gut homing phenotype through upregulation of specific adhesion molecules. T cells imprinted for small intestinal homing express integrin α4β7, α4β1, β2 integrins and CCR9, while cells primed for migration to the colon show high levels of integrin α4β7 and GPR15 (5–8). Upon recirculation, these T cell subsets may subsequently migrate to the gut as their target tissue along chemotactic gradients, where they interact with the molecules expressed by endothelial cells to initiate the multistep extravasation process of gut homing. Tethering and rolling mediated by low-affinity binding of selectins (predominantly L-selectin) and integrins (α4β7, α4β1) on T cells to their ligands expressed on endothelial cells (GlyCAM-1, mucosal addressin cell adhesion molecule-1 (MAdCAM-1), vascular cell adhesion molecule 1 (VCAM-1), respectively) slow the cells down to increase availability for activation by tissue-secreted chemokines (e.g. CCL25, CXCL10) (9). This leads to conformational changes of the integrins and, hence, to firm interaction of integrins with cell adhesion molecules and subsequent arrest of activated T cells, followed by transmigration through the endothelium into the tissue. Upon arrival at the site of action, T cells adapt their make-up of surface molecules to their environment (e.g., upregulation of integrin αEβ7) leading to retention in the tissue or, if not activated, recirculation to the blood and lymph (e.g., *via* S1PR/S1P) (10, 11).

**Figure 1:**
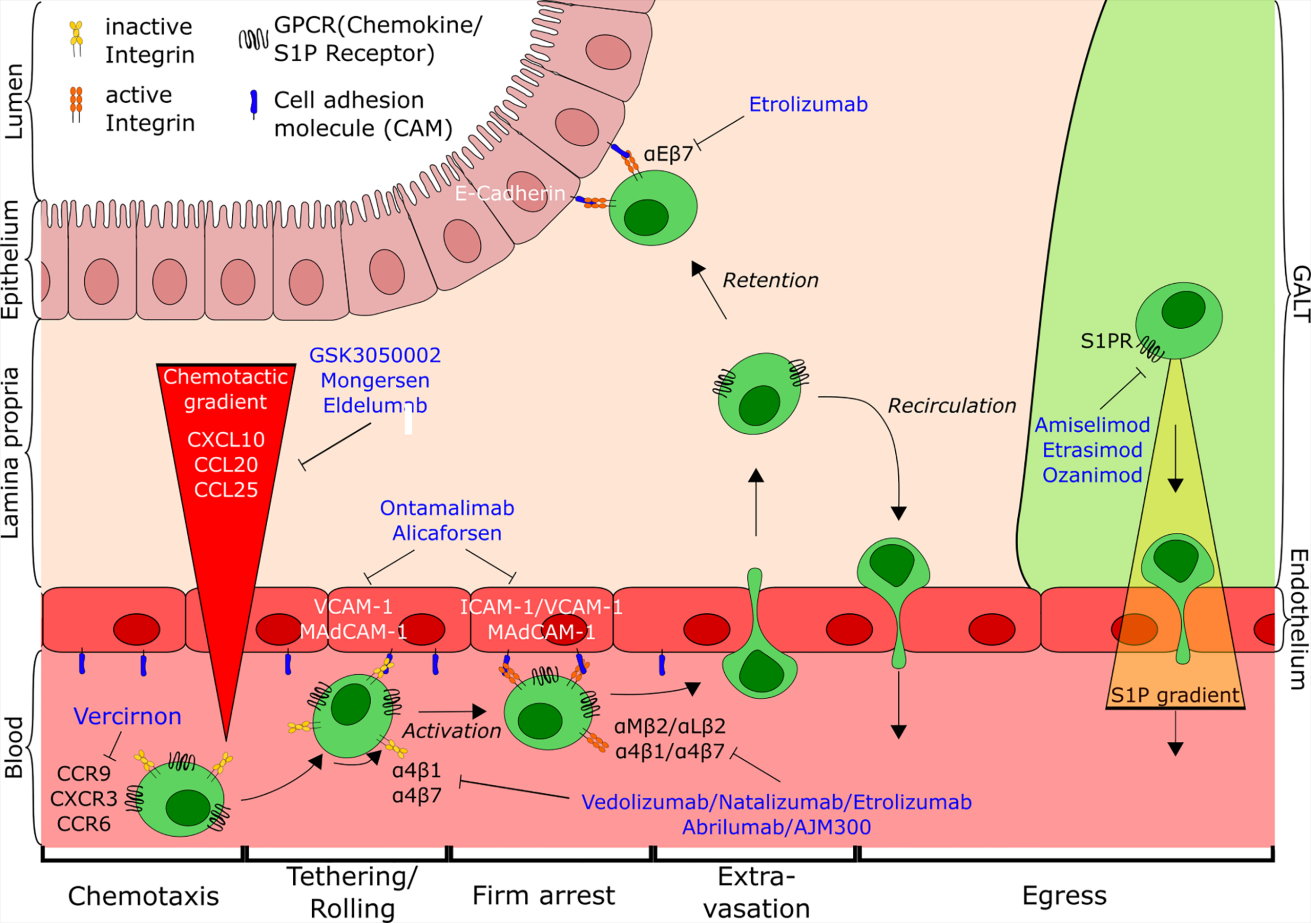
Overview of T cell trafficking in the intestine indicating the points of action of current and potential future anti-trafficking agents for the treatment of IBD. Tethering and rolling of cells on the endothelial wall mediated by interaction of low-affinity integrins with their respective ligands (e.g. α4β7-MAdCAM-1) leads to the exposure to a chemokine gradient (CCL25, CXCL10, CCL20). Subsequent activation of cells causes conformational changes of the integrins, followed by firm arrest and extravasation of T cells to the gut. There, cells are either retained in the tissue through interaction with the epithelium (αEβ7-E-cadherin) or antagonism of egress, or recirculate into the blood from gut and GALT along the S1P-gradient. CD, cluster of differentiation; CCR, Chemokine receptor; CXCR, CXC-motif chemokine receptor; GPCR, G-protein coupled receptor; S1P, Sphingosine-1-phospate; S1PR, Sphingosine-1-phosphate receptor; ICAM-1, Intercellular adhesion molecule 1; VCAM-1, Vascular cell adhesion molecule 1; MAdCAM-1, Mucosal addressin cell adhesion molecule-1; GALT, Gut-associated lymphoid tissue.

T cell trafficking has emerged as one of the hallmarks of IBD pathogenesis and as a potential goldmine for a plethora of new treatment options for IBD by targeting the different steps of this process. This mini-review aims to provide a comprehensive overview of current and future therapeutics based on interference with T cell trafficking, highlighting their mechanisms and potential to reduce disease burden (**Figure 1**).

## Targeting Cell Adhesion Molecules – Blockade on the Side of the Effector Tissue

Cell adhesion molecules expressed by effector tissues are major mediators of T cell recruitment and intestinal inflammation and serve as promising targets for therapeutic anti-trafficking strategies.

Already in the 1990s, selectively blocking the interaction of β2 integrins with intercellular adhesion molecule 1 (ICAM-1) using antibodies against CD18/ICAM-1 or ICAM-1 antisense oligonucleotides showed promise by reducing inflammation and cell infiltration in 2,4,6-trinitrobenzenesulfonic acid (TNBS)-colitis in rats (12), dextran sodium sulfate (DSS) colitis in mice (13) or acetic acid-induced inflammation in rats (14). Expression of ICAM-1 is upregulated by endothelial cells under inflammatory conditions (13, 15), which leads to increased extravasation of leukocytes (e.g., neutrophils and T cells) expressing β2 integrins. In 1998, Yacyshyn and colleagues could demonstrate that the ICAM-1 antisense oligonucleotide ISIS 2302/alicaforsen administered intravenously was well tolerated and showed promising results for the treatment of CD (16). Treatment with alicaforsen reduced expression of ICAM-1 on high endothelial venules (HEV), thereby hindering leukocyte extravasation. However, two subsequent trials with alicaforsen in active CD could not demonstrate superiority over placebo (17, 18). Alicaforsen was also investigated as an enema for topical application in the treatment of UC and pouchitis. Initial clinical evaluations showed improved clinical scores for both diseases (19, 20). However, later studies in mild-to-moderate UC failed to reach their primary endpoints (21, 22). A phase III trial with alicaforsen enema for the treatment of pouchitis patients refractory to antibiotics was completed last year. The treatment with alicaforsen was safe and even though the primary endpoint of endoscopic remission at week 10 showed no difference between treatment with alicaforsen and placebo, the portion of patients reporting a reduction of stool frequency was higher in the alicaforsen compared with the placebo group (NCT02525523).

Another important cell adhesion molecule involved in gut homing and upregulated upon inflammation is VCAM-1. VCAM-1 antagonists proved superior to ICAM-1 and MAdCAM-1 blockade in the murine model of DSS colitis (23), and the monoclonal anti-α4 integrin antibody natalizumab has been successfully used for blockade of VCAM-1-dependent leukocyte trafficking in patients with active CD (24–27). However, due to the ubiquitous expression of VCAM-1, systemic blocking of the VCAM-1 homing cascade was associated with severe adverse events like progressive multifocal leukoencephalopathy (PML) (28, 29), underscoring the need for gut-selective targeting of T cell trafficking. Therefore, although VCAM-1–α4β1 is strongly involved in small intestinal T cell recruitment (30), it is questionable, whether targeting VCAM-1 is a promising target for the treatment of IBD.

Ontamalimab (formerly SHP647) is an antibody binding MAdCAM-1, the ligand of α4β7 integrin and L-selectin. MAdCAM-1 is predominantly expressed on HEVs of the gut and GALT (31) and its expression is strongly induced by TNF-α under inflammatory conditions and in IBD patients (32–34). Pre-clinical trials with the murine anti-MAdCAM-1 antibody MECA-367 demonstrated reduced lymphocyte recruitment to the gut and reduction of inflammation in the T cell transfer colitis model in Scid mice (35, 36). A first human phase I study could show safety of anti-MAdCAM-1 therapy in patients with active UC and a change of biomarkers compared to baseline (37), and efficacy in the treatment of UC was confirmed in a phase II trial (TURANDOT) (38). In a phase II trial for the treatment of moderate-to-severe CD (OPERA) clinical endpoints did not reach statistical significance in comparison to placebo (39, 40), but treatment led to a reduction of soluble MAdCAM-1 and to an increase of circulating β7^+^ central memory T cells and elevated CCR9 gene expression (41). The phase III trials for ontamalimab in both UC and CD were discontinued following a take-over of the developing company (42). However, ontamalimab remains a promising therapeutic agent. Treatment did not lead to central nervous system complications and induced very low levels of anti-drug antibodies (43). With regard to L-selectin as an additional interaction partner of MAdCAM-1, ontamalimab might not just be an imitation of anti-α4β7 antibody therapy, but dispose over a unique mechanism of action (44, 45). Furthermore, expression of MAdCAM-1 in other mucosal tissues as well as in joints, eyes, skin and liver (46–48) make it a potential treatment option for extraintestinal manifestations of IBD and other inflammatory diseases (49).

Taken together, these data show the potential of targeting cell adhesion molecules in the treatment of IBD, especially in selected subgroups of patients, and suggest that, despite some deceptions and obstacles, it seems worth further developing respective compounds.

## Blockade of Chemokines – Reducing Leukocyte Attraction

In addition to cell adhesion molecules, chemokines play a pivotal role in T cell recruitment to the gut and offer another approach for therapeutic targeting.

CCL25 is a chemokine expressed in the small intestine under homeostatic conditions and strongly upregulated in the ileum and also the colon upon inflammation (50–52). Its receptor CCR9 is found on T cells imprinted for gut homing (53–56). Even though CCR9 is highly expressed on regulatory T cells (Treg) and plays a leading role in establishing self-tolerance in the thymus (57), the CCR9-CCL25 axis has been implicated in inflammation, especially of the small intestine (52). Isolated CCR9^+^ T cells from CD patients show markedly higher expression of IL17 and IFNγ upon stimulation compared to controls (58), and stimulation of T cells through CCR9 leads to activation of α4β1 and α4β7 integrins and, hence, increased extravasation (59, 60).

Blocking either CCR9 or CCL25 in mice treated with TNFα or in the SAMP1/YitFc model of ileitis demonstrated reduction of leukocyte migration to the small intestine and strong inhibition of inflammation (61, 62). The oral CCR9 antagonist CCX282-B/vercirnon was successfully used in the TNF^ΔARE^ ileitis mouse model (63) as well as for the treatment of moderate-to-severe CD in a phase II study (64). A subsequent phase III study failed to demonstrate efficacy of vercirnon as induction therapy (65, 66). Data from animal models and patients show a strong homeostatic role for the CCR9-CCL25 axis in the small intestine, but a clear association with inflammation in the colon (66, 67) and a study depleting CCR9^+^ cells through leukapheresis (68) showed promising results, suggesting that blocking CCR9-CCL25 interaction might be an option for the treatment of UC.

Another chemotactic stimulus for gut infiltration of T cells is CXCL10. CXCL10 expression is induced by IFNγ (69) and markedly upregulated in colitis (70, 71). Its receptor CXCR3 is found on effector T cells, β7^+^ peripheral blood mononuclear cells (PBMCs), lamina propria mononuclear cells (LPMCs) and intraepithelial lymphocytes (IELs) and a high number of CXCR3^+^ cells can be found in biopsies from UC and CD patients (72). Treatment with anti-CXCL10 antibodies attenuated colitis in IL10-deficient mice and in DSS colitis and reduced cell infiltration to the lamina propria (73–76). In clinical studies, treatment with a monoclonal antibody against CXCL10 was efficient for the treatment of rheumatoid arthritis (MDX-1100) (77). However, blocking CXCL10 with the antibody eldelumab failed to induce remission in patients with moderate-to-severe UC or CD (78–80). Still, in subgroups of anti-TNFα naïve patients, CXCL10 blockade ameliorated mucosal response, suggesting that this treatment could be effective in selected patients (66).

CCL20 is a chemokine implicated in both inflammation and homeostasis and is predominantly expressed by mucosal epithelial cells (81). CCL20 expression is induced through TNFα and elevated in CD patients (82). In pre-clinical studies, neutralization of CCL20 reduced T cell infiltration and attenuated colitis in the murine TNBS-model (83). Bouma and colleagues reported a dose-dependent decrease of cells bearing CCR6, the receptor for CCL20, in healthy human volunteers after treatment with the humanized antibody GSK3050002 against CCL20 (84). However, to our knowledge, no CCL20 antagonist has been used in clinical trials of IBD so far. Yet, mongersen, an oral Smad7 anti-sense oligonucleotide indirectly regulates CCL20 expression. Smad7 is highly expressed in the mucosa of IBD patients and acts as an inhibitor of TGFβ1 signalling, an important negative regulator of TNFα signalling. Consistently, blocking Smad7 expression through the administration of an anti-sense nucleotide restored TFGβ1 signalling (85). In the TNBS and oxazolone colitis mouse models, treatment with a Smad7 anti-sense oligonucleotide led to reduction of inflammation (86). Treatment of CD organ explants with mongersen reduced Smad7 and CCL20 expression and serum levels of CCL20 in patients responding to mongersen were significantly reduced (82). In 2015, Monteleone and colleagues reported significantly higher response and remission rates after treatment with mongersen compared to placebo in patients with active CD (87). However, a subsequent phase III study published last year failed to demonstrate efficacy for the treatment of CD (88). Data to interpret these results with regard to the indirect effect on CCL20 are lacking.

GPR15 is a recently deorphanized receptor expressed on a large subset of colon-homing T cells. GPR15 is found on Foxp3^+^ Treg cells in mice and important for the maintenance of large intestinal homeostasis, while data from humans suggest higher expression on effector T cells (7, 89, 90). The ligand for GPR15 (GPR15L) is expressed by epithelial cells in the colon and the skin and chemotactic abilities have been reported (91), suggesting that the GPR15-GPR15L axis might be a potential target for modulating intestinal inflammation.

Collectively, these studies show the large potential of treating IBD by targeting chemokines and their receptors, but also indicate that interfering with chemokine signalling seems to be a complex approach that has not resulted in the approval of therapeutic agents so far.

## Targeting Trafficking on the T Cell Side – A Story of Success

The prime example for successful treatment of IBDs by targeting leukocyte trafficking is the anti-α4β7 integrin antibody vedolizumab (92–97). Binding to its target leads to the internalization of the α4β7 integrin, inhibiting the interaction with its ligand MAdCAM-1, which is virtually exclusively expressed on the endothelium of the gut and GALT (31, 98, 99). This very selective and highly gut-specific mode of action leads to reduced intestinal lymphocyte counts and inflammation, while retaining an excellent safety profile with few side effects (100–102). The gut specificity is also thought to account for the safety profile advantage over broader α4 integrin blockade by the antibody natalizumab, which, while being effective for the treatment of preclinical cotton-top tamarin colitis (103) and active CD (24, 25), was withdrawn from widespread use after several cases of PML (28, 29).

The example of vedolizumab paved the way for the current development of additional drugs with a similar mode of action. Abrilumab, another anti-α4β7 integrin antibody that is subcutaneously administered, successfully completed phase II trials for moderate-to-severe UC (104–106). Moreover, the oral small molecule α4 integrin inhibitor AJM300 successfully attenuated inflammation and cell infiltration in the adoptive T cell transfer colitis model (107) and currently undergoes phase III testing in UC. While no cases of PML were observed in phase II trials, it will be important to thoroughly investigate the safety profile of AJM300 in further studies, since it is likely that it affects central nervous immune surveillance similar to natalizumab, although it might have a favorable pharmacological profile (108, 109).

## Integrin Blockade Beyond α4β7-Blockade – Interfering With Retention

Aiming to expand the clinically successful anti-α4β7 strategy, the humanized monoclonal antibody etrolizumab was developed to target the β7-subunit of α4β7 as well as αE (CD103) β7 integrin hetereodimers (110). CD103 expression on T cells is induced by T cell receptor signalling and TGFβ, which is released by several cellular sources in the intestine (111, 112). αEβ7-expressing cells are able to interact with epithelial (E-) cadherin expressed by intestinal epithelial cells (IECs) and may thereby be retained in the tissue (113). Furthermore, evidence from cancer and gastritis research suggests that this interaction serves as a costimulatory factor for T cell receptor activation in CD8^+^ and CD4^+^ T cells, respectively (114–117). Despite CD103 expression being associated with a Treg phenotype in mice (118–120), recent evidence suggests a pro-inflammatory Th1, Th17 and Th1/17 phenotype for αEβ7^+^ CD4^+^ T cells with reduced expression of Treg markers in the large intestine of UC patients, proposing a role for these cells in disease pathobiology (121). Furthermore, the role of CD4^+^ tissue resident memory T cells (Trm), which can also express CD103, in human IBD and murine models of colitis has recently been highlighted (122). The data suggested involvement of these cells in the development of IBD flares and as a switch-point for experimental colitis further substantiating the potential of αEβ7 as a therapeutic target (122). With promising results from the adoptive transfer colitis mouse model (123) and phase II trials, large-scale phase III programs were launched for etrolizumab in active UC and CD. In recently presented data from the UC trials, etrolizumab, while being well tolerated, only met the primary endpoint in two out of three induction studies and in none of the two maintenance studies (124–126). Despite these discouraging results, several key secondary endpoints were met and, strikingly, numerically similar clinical and endoscopic outcomes were reported for etrolizumab and the anti-TNFα antibodies infliximab and adalimumab (127, 128), thus supporting biological activity of etrolizumab. Further analyses, including the previously suggested ability of CD103 to predict response to therapy (129) are eagerly awaited. Moreover, the pivotal CD phase III trial program is nearing its completion and experimental evidence indicates that αEβ7 integrin might be even more important in that context. Specifically, in line with previous reports, Ichikawa, Lamb and colleagues demonstrated an increased abundance of CD103^+^ cells in the ileum compared to the colon (130), suggesting that ileal CD might be a particularly promising entity to treat with etrolizumab. This is further supported by the observation that α4β7 blockade alone did not sufficiently reduce homing of CD patient-derived effector T cells to the ileum in an adoptive transfer model (131).

Taken together, in spite of disappointing results in the UC phase III trials, books should not be closed prematurely over etrolizumab, especially regarding subsets of patients with increased CD103 expression or ileal disease location.

## Sequestration of Cells in Lymph Nodes by S1PR Modulators

Cellular retention cannot only be modulated by interfering with tissue anchorage, but also by modifying exit cues. This is the principle of the emerging field of S1P modulation. Physiologically, recirculation of T cells from the tissue to the blood is mediated by a constantly generated S1P gradient, with high concentrations in the blood. Low concentrations in tissues are upheld by enzymatic degradation of S1P by the S1P-lyase (132–134). S1P is sensed by S1P receptors (S1PR)1-5, which internalize on ligand binding, thereby inducing transient tissue retention and providing the opportunity for activation and antigen sensing of retained cells (135–137). Furthermore, it has been demonstrated that activation-induced CD69 directly interacts with S1PR1 and leads to its removal from the cell surface, thereby contributing to tissue retention of activated T cells (138). S1P modulation for IBD therapy aims to sequester naïve and central memory T cells in lymphoid tissues, inducing circulatory lymphopenia and thereby cutting off the supply of potentially pathogenic T cells migrating to the site of inflammation (11). Generally, S1PR modulators for IBD treatment are agonists, which can be distinguished by differential selectivity for S1PRs.

After showing promising result in preclinical models (TNBS colitis in rats and adoptive transfer colitis in Scid mice) (139) and after successfully completing a phase II study in moderate-to-severe UC (140), first results from phase III trials of the oral S1PR1- and S1PR5-selective agonist ozanimod for the treatment of moderate-to-severe UC were presented recently. Ozanimod demonstrated statistically significant improvements in clinical remission and response, endoscopic and mucosal healing without raising safety issues and can therefore be considered to enter the treatment algorithm for UC in the future (141, 142). In the adoptive transfer colitis model, etrasimod, a S1PR1, 4, 5 agonist, was able to reduce inflammation (143). After successful completion of phase II trials, etrasimod recently entered phase III testing for moderate-to-severe UC (144). In contrast, a clinical trial of the selective S1PR1 modulator amiselimod as a treatment for CD could not demonstrate an impact on clinical or biochemical disease activity, despite promising results in a preclinical study employing the adoptive T cell transfer model of chronic colitis (145, 146).

Taken together, S1PR modulation proved to be a potent tool for the treatment of IBD, but different results observed with agonists with differential selectivity highlight the complexity of this approach and therefore warrant further research.

## Discrepancies Between Mouse And Man – Challenges in Drug Development

As referenced at several points of this review, promissing preclinical observations have promted clinical trials (**Table 1**). However, not all insights from preclinical studies have been reproducable in humans. Reasons for late stage failures in drug development despite preclinical efficacy and often clear indications of biological effects in humans are manifold. Animal models are a valuable and indispensable tool to uncover disease pathogenesis and mechanisms underlying therapeutic effects (3, 134) and advances in medical research today would not be on the same level without insights from such model systems. However, mouse models of intestinal inflammation cannot fully reproduce the complexity of IBD as a multifactorial disease in certain aspects. In recent years, the importance of the microbiome in IBD was highlighted by several studies (147–150). The controlled environments in animal facilities limit microbiome diversity in experimental animals, but can differ between facilities and therefore can influence results (151). Specific-pathogen-free (SPF) environments in preclinical setups limit the predictability of adverse events related to infections (e.g. PML). These apparent limitations can also be considered as a strength, as comparable housing and nutrition enables researchers to uncover the influence of microbiota on disease pathology (152). Furthermore, IBD pathomechanisms are portrayed only partially, e.g. DSS-induced colitis is driven by the innate immune system and is induced even in the absence of lymphocytes (153–155), whereas pathology in the T cell transfer colitis model is induced by the transfer of naïve T cells to immunodeficient hosts (156, 157). Therefore, different models can produce contradicting results for the same treatment, as it is, for instance, the case for anti-α4β7 blockade in acute DSS-induced and T cell transfer colitis (158). On the other hand, this cell specific mode of action can help to unravel the contribution of different cell types to disease processes and lead to a better understanding of therapeutic mechanism (122).

**Table 1:** Anti-trafficking agents used in the clinic or still in development with details on their specific target, mode of action and preclinical and clinical efficacy.

Target	Origin	Drug	Mode of action	Preclinical Data			Clinical Data		**Observed effects**
				**Model**	**Effective?**	**Disease**	**Administration**	**Primary endpoint(s) met?**	
ICAM-1	Endothelium	Alicaforsen	Anti-sense oligonucleotide	DSS colitis mouse model (13)	Yes	CD (Phase II) (16)	IV	No	Steroid withdrawal
						CD (Phase II) (17)	IV	No	Steroid withdrawal
						CD (Phase II) (18)	IV	No	N/A
						UC (20)	Enema	Yes	Clinical response
						Pouchitis (NCT02525523)	Enema	Not yet published	Reduced stool frequency
MAdCAM-1	Endothelium	Ontamalimab	Monoclonal antibody	Adoptive T cell transfer colitis mouse model (35,36)	Yes	UC (Phase I) (37)	IV/SC	No	Changes in biomarkers compared to baseline
						UC (Phase II) (38)	SC	Yes	Clinical remission
						CD (Phase II) (39,40)	SC	No	Clinical remission in patients with higher endoscopic activity
						UC and CD (Phase III)	SC	Discontinued	
CCR9	Lymphocytes	Vercirnon	Small molecule antagonist	TNF^ΔARE^ ileitis mouse model (62)	Yes	CD (Phase II) (64)	PO	Yes	Clinical response, remission and steroid-free remission
						CD (Phase III) (65)	PO	No	N/A
CXCL10	Epithelium	Eldelumab	Monoclonal antibody	IL-10^-/-^ and piroxicam colitis mouse model (74)	Yes	UC (Phase II) (78)	IV	No	Improvement of IBDQ score
				DSS colitis mouse model (75)	Yes	UC (Phase II) (80)	IV	No	N/A
				IL-10^-/-^ colitis mouse model (76)	Yes	CD (Phase II) (79)	IV	No	Numerically higher remission and response rates
Smad7	Epithelium	Mongersen	Anti-sense oligonucleotide	TNBS and oxazolone colitis mouse model (86)	Yes	CD (Phase II) (87)	PO	Yes	Clinical remission and response
						CD (Phase III) (88)	PO	No	N/A
α4 integrin	Lymphocytes	Natalizumab	Monoclonal antibody	Cotton-top tamarin colitis model (103)	Yes	CD (24)	IV	Yes	Clinical remission and response
						CD (25)	IV	Yes	Clinical remission and response
						CD (Phase III) (26)	IV	Yes	Clinical response
						CD (27)	IV	Yes	Clinical response
		AJM300	Small molecule antagonist	Adoptive T cell transfer colitis mouse model (107)	Yes	UC (Phase II) (109)	PO	Yes	Clinical remission and mucosal healing
α4β7 integrin	Lymphocytes	Vedolizumab	Monoclonal antibody	Cotton-top tamarin colitis model (96)	Yes	UC (Phase III) (94)	IV	Yes	Clinical response, remission and mucosal healing
						CD (Phase III) (97)	IV	Yes	Clinical response, remission and steroid-free remission
		Abrilumab	Monoclonal antibody	N/A		UC (Phase II) (104)	SC	Yes	Clinical response, remission and mucosal healing
						UC (Phase II) (105)	SC	Yes	Numerically higher remission, response and mucosal healing rates
β7 integrin	Lymphocytes	Etrolizumab	Monoclonal antibody	Adoptive T cell transfer colitis mouse model (123)	Yes	UC (Phase II) (110)	SC	Yes	Clinical remission
						UC (Phase III) (124, 125, 127)	SC	Yes/No	Clinical response and endoscopic improvement
						CD (Phase III)	SC	Ongoing	
S1PR	Lymphocytes	Ozanimod (S1PR1/5)	Small molecule agonist	TNBS colitis rat model and adoptive T cell transfer colitis mouse model (139)	Yes	UC (Phase II) (140)	PO	Yes	Clinical response and mucosal healing
						UC (Phase III) (141, 142)	PO	Yes	Clinical remission, response and mucosal healing
		Etrasimod (S1PR1/4/5)	Small molecule agonist	Adoptive T cell transfer colitis mouse model (143)	Yes	UC (Phase II) (144)	PO	Yes	Clinical remission, response and histological improvement
		Amiselimod (S1PR1)	Small molecule agonist	Adoptive T cell transfer colitis mouse model (146)	Yes	CD (145)	PO	No	Reduced lymphocyte counts

IV, intravenous; SC, subcutaneous; PO, per os; UC, ulcerative colitis; CD, Crohn's disease; N/A, not available.

Moreover, GPR15 expression was previously reported to direct Treg cells to the large intestine and defects in GPR15 led to increased susceptibility to colitis in a *Citrobacter rodentium* infection model and reduced suppression or rescue of inflammation in anti-CD40 and T cell transfer colitis models (7). In contrast, Nguyen and colleagues demonstrated GPR15 expression on murine Th1 and Th17 cells in addition to Treg cells and a GPR15 dependency in the induction of colitis in the T cell transfer model, thus further highlighting the potential of differential outcomes even when working with the same receptor in different setups (159). The same study also highlighted another reason for potential species discrepancies: in contrast to GPR15 expression on murine Th1, Th17 and Treg cells, expression of GPR15 was associated with a Th2 phenotype in the large intestinal lamina propria of UC patients. This observation was attributed to species-specific enhancer sites binding GATA3, the Th2 lineage defining transcription factor, in the human GPR15 gene, which are absent in the mouse genome. Species differences between mouse and man have also been reported for other potential targets of investigated drug candidates, including CD103 (118–121). Inadequate experimental design can further be the cause for limited reproducibility. Therefore, many groups have developed concepts to improve the quality of animal studies (e.g., by using completely randomised experimental designs or by conducting experiments at a similar time of day) (160–162). Finally, several of the compounds reviewed here showed promising results in phase II trials but failed to reach primary endpoints in phase III studies (see **Table 1**). Possible explanations could be the stricter definition of primary endpoints in phase III trials [e.g. mongersen (87, 88)], or differences in patient cohorts or study design [e.g. vercirnon (64, 65)].

Taken together, these aspects demonstrate the complexity and importance of preclinical testing in IBD anti-trafficking agent development underscoring the need for careful evaluation of different model systems as well as systematic analysis of potential species differences for successful translation of preclinical findings to the clinic.

## Concluding Remarks

The implication of intestinal T cell trafficking in the pathogenesis of IBD is undisputed. Targeting associated either on endothelial/epithelial cells or on the circulating T cells has proven to hinder cell infiltration effectively. However, the important role of T cell recruitment for tissue homeostasis and pathogen defence underscores the need for selective inhibition strategies to ensure the safety of the therapeutic agent. Discrepancies between human and murine physiology (e.g. GPR15, CD103 expression) need to be carefully evaluated, when translating preclinical findings into clinical treatment options. Despite being outside the scope of this mini-review, the therapeutic options discussed may also affect trafficking of other immune cells that need to be taken into account. And, finally, development of further and a more detailed understanding of approved therapeutic options can only be the first step. Regarding the substantial portion of patients showing primary or secondary non-response, individualized treatment strategies to predict and optimize therapeutic outcomes are an important unmet need. However, advances in the field of T cell trafficking might also contribute to solutions to that problem.

## Author Contributions

MW, EB, and SZ wrote the manuscript. All authors contributed to the article and approved the submitted version.

## Funding

German Research Foundation (DFG, ZU 377/4-1, TRR241 C04), Interdisciplinary Center for Clinical Research (IZKF) of the University Erlangen-Nuremberg (A84).

## Conflict of Interest

The authors declare that the research was conducted in the absence of any commercial or financial relationships that could be construed as a potential conflict of interest.
